# Evaluation of Sensibility Threshold for Interocclusal Thickness of Patients Wearing Complete Dentures

**DOI:** 10.1155/2017/5138950

**Published:** 2017-06-18

**Authors:** Kujtim Sh. Shala, Linda J. Dula, Teuta Pustina-Krasniqi, Teuta Bicaj, Enis F. Ahmedi, Zana Lila-Krasniqi, Arlinda Tmava-Dragusha

**Affiliations:** Department of Prosthetic Dentistry, School of Dentistry, Faculty of Medicine, University of Pristina, Pristina, Kosovo

## Abstract

**Objective:**

The aim of this study was to evaluate sensibility threshold for interocclusal thickness in experienced and nonexperienced denture wearers after the insertion of new complete dentures.

**Materials and Methods:**

A total of 88 patients with complete dentures have participated in this study. The research was divided into two experimental groups, compared with the previous experience prosthetic dental treatment. The sensibility threshold for interocclusal thickness was measured with metal foil with 8 *μ*m thickness and width of 8 mm, placed between the upper and lower incisor region. Statistical analysis was performed using standard software package BMDP (biomedical statistical package).

**Results:**

Results suggest that time of measurement affects the average values of the sensibility threshold for interocclusal thickness (*F* = 242.68, *p* = 0.0000). Gender appeared to be a significant factor when it interacted with time measurement resulting in differences in sensibility threshold for interocclusal thickness (gender: *F* = 9.84, *p* = 0.018; *F* = 4.83, *p* = 0.0003).

**Conclusion:**

The sensibility threshold for interocclusal thickness was the most important functional adaptation in patient with complete dentures. A unique trait of this indicator is the progressive reduction of initial values and a tendency to reestablish the stationary state in the fifteenth week after dentures is taken off.

## 1. Introduction

The high tactile sensibility of periodontium, the oral mucosa and muscles, is important for the regulation and controls the masticatory and occlusal forces. Knowledge of this property of support tissue is of great importance for the recognition of bruxism, traumatic constipation, and oral dysfunction. Clinical observations show that small changes in occlusion may affect the functional state of the masticatory efficiency system and act as a factor in forming par function [1–5]. Specifically, an increase in the value of the sensibility threshold for interocclusal thickness after an optimal period of neuromuscular adaptation suggests the presence of obstacles in the level of occlusal surfaces.

The following study builds upon previous studies which have looked at the importance of the edentulous patients that lack several oral functions, such as masticatory efficiency and bite force, as they have no periodontal receptors [6, 7]. Manly et al. [3] in their research have found that edentulous patients and subjects with intact dental arches can only estimate the thickness of the disc situated between dental arches. The capacity of dentate subjects to discriminate the thickness of objects placed between the teeth seems to depend on receptors in the periodontal ligament and temporomandibular joint and muscles [8]. Since periodontal receptors are highly sensitive to small deformations of periodontal fibers, it is assumed that these receptors may be responsible for determining the thickness of interocclusal facilities. Periodontal receptors are also regulatory and balancing biting forces. For example, they participate in the control of interocclusal perception. Some studies have also shown that, in cases where the receptors in temporomandibular joint (TMJ) and periodontal receptors are disconnected, information of the sensitivity of the receptors which are found in masticator muscles contributes to the evaluation of the thickness of the objects between the teeth [9].

Siirilä and Laine [9] did not encounter any differences to natural teeth in size of sensibility threshold for interocclusal thickness between frontal and lateral teeth. Kawamura and Majima [10] describe the decrease of sensibility threshold for interocclusal thickness from incisive teeth up to molar, while Loewenstein and Rathkamp [11] and Nishiyama et al. [12] think that the antagonist of central incisors is more sensitive in the perception of interocclusal thickness. Research undertaken in order to determine the ability of the patient to differentiate the degree of hardness of the material tested, immediately after wearing complete dentures and after 7 days, has confirmed that a subject had been able to identify the degree of hardness of the material [13].

The aim of this study was to evaluate sensibility threshold for interocclusal thickness in experienced and nonexperienced denture wearers after the insertion of new complete dentures.

## 2. Materials and Methods

A total of 88 patients with complete dentures have participated in this study. The research has been accepted and approved by the Institutional Ethic Committee (School of Dental Medicine, University of Pristina) and a written consent was obtained from each subject.

The main criterion for inclusion in the investigation was patients with Angle I relationship between upper and lower jaw and where alveolar ridge has been reduced uniformly. Exclusion criteria in the study were patients of age over 70 years and with orthodontic bite anomalies in sagittal and transverse plane, dysfunctions of the temporomandibular joint, and high rate of resorption of the alveolar ridge (negative alveolar ridge).

The research was divided into two experimental groups, compared with the previous experience prosthetic dental treatment. Group 1 consisted of patients who for the first time take (wearing) maxillary and mandibular complete dentures; Group 2 consisted of patients who have previous experience and who even earlier had complete dentures.

The patients were selected at the Department of Prosthodontics, Dental School, Faculty of Medicine, University of Pristina in Kosovo. Clinical procedures for complete denture fabrication were as follows: we made preliminary impression using a stock tray with the irreversible hydrocolloid in lower and upper residual ridge. Final impressions were taken with custom tray. The polyether material is syringed around the borders of the tray until they are all covered for lower and upper jaw. After that, we were ready to take final impression with silicone impressions material. In the third meeting we confirmed fit and extension, contour wax rims for lip support, future incisal edge position, occlusal plane, occlusal vertical dimension, and midline. We selected tooth mold, tooth shade, and desired occlusal scheme. In the try-in appointment we checked clinical observation of tooth contacts, protrusive and lateral relations, controlling factors of movement, vertical occlusal dimension, phonetics, and esthetics. We performed modifications as necessary. In the final appointment we completed the rehabilitation of patient with the insertion of the complete dentures. Typical postinsertion follow-up includes 24-hour, one-week, and one-month appointments.

After insertion of new complete dentures, all patients were tested for sensibility threshold for interocclusal thickness. The measurements were done by a single examiner to reduce interobserver error, and each measure was taken six times for six months. The thickness perception test was repeated on days after insertion of new complete dentures, 2–5 weeks, 3–10 weeks, 4–15 weeks, 5–10 weeks, and 6–25 weeks after the insertion of new complete dentures.

**Figure 1:**
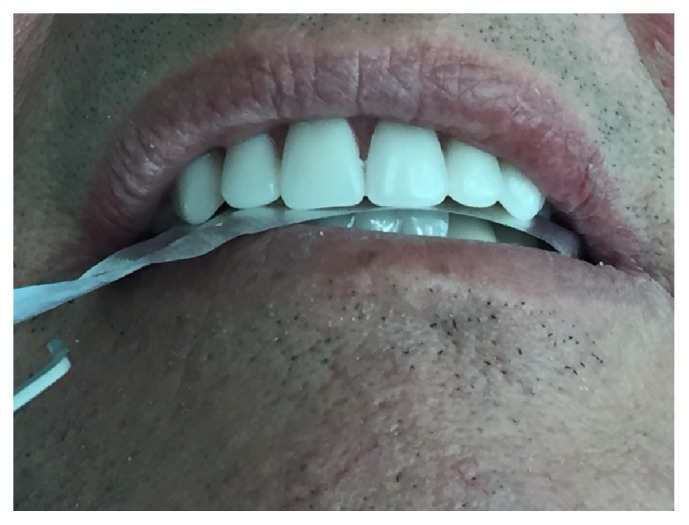
Metal foil placed between the upper and lower incisor region.

The sensibility threshold for interocclusal thickness was measured with metal foil (Shimstock-Metallfolie, 8 *μ*m thickness and width of 8 mm, which does not expand and will burst during the bite, Hanel-Medizinal, Nürtingen, Germany), placed between the upper and lower incisor region in incisal edge-to-incisal-edge position teeth (edge-to-edge bite) (Figure 1).

The patient was invited to sit on a chair in a quiet room and was instructed on how to perform the test. The purpose of testing was explained to the patients and the patients were required to not touch the metal foil with their tongue and were asked whether they feel the presence of a foreign body or not. Since it was purpose subjective method of objectification response, the procedure of applying the foil was made with eyes closed, so that patients at any moment were not sure about whether real foil was applied or not.

The test began with the initial thickness of metal foil of 150 *μ*m being successively reduced to 30 *μ*m. When the patient stated that he/she most felt the presence of a foreign body between teeth, the thickness was increased to 8 *μ*m. First perception detected from patients was taken as individual sensibility threshold for interocclusal thickness. During follow-up, the initial thickness was smaller but on the whole greater than the expected. Over the evaluation of the results obtained, the value of the test sensibility has been higher than the threshold set for the thickness of the foreign body in the occlusal surface of complete dentures.

### 2.1. Data Analysis

Statistical parameters of processing results include all levels: from description data to statistical conclusions to model the behavior of factors, which are used for research of functional assessment of therapeutic effect of complete dentures.

Statistical analysis was performed using standard software package BMDP (biomedical statistical package), dedicated to research in the biomedical sciences, which includes all methods of statistical procedures (Dixon, 62). Testing parametric data were done with One-Way Repeated Measurement of ANOVA test. The study results are presented in table and graphic form.

## 3. Results

A total of 88 patients with complete dentures participated in this study. There were 42 females and 46 males, and the average age was 55.2 with a standard deviation of 5.72. Of these, forty-five patients belonged to the nonexperienced group that wore new complete dentures for the first time, while forty-three patients had been wearing complete dentures for a while (the experienced group) (Table 1).

**Figure 2:**
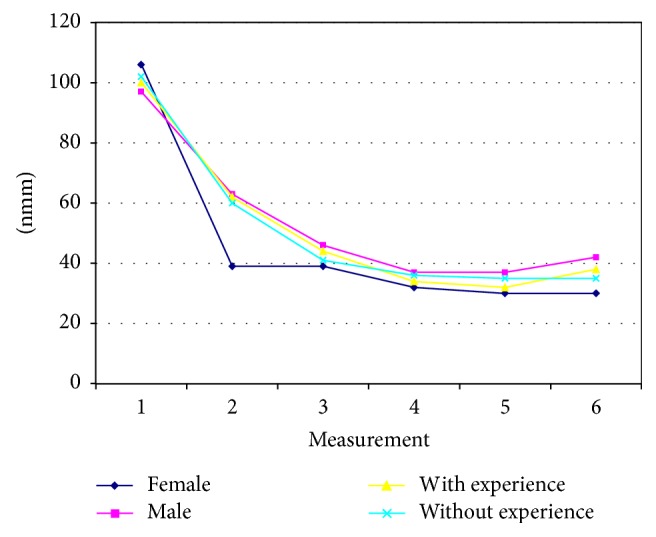
Dynamics of sensibility threshold for interocclusal thickness (gender and experience with complete dentures).

The average values of the sensibility threshold for interocclusal thickness in the experienced and the nonexperienced groups were measured six times over a period of six months (Table 2). Results suggest that time of measurement affects the average values of the sensibility threshold for interocclusal thickness (*F* = 242.68, *p* = 0.0000). During the first four measurements, the values fall consistently and reach a stationary condition on the fourth measurements (Figure 2).

Gender appeared to be a significant factor when it interacted with time measurement resulting in differences in sensibility threshold for interocclusal thickness (gender: *F* = 9.84, *p* = 0.018; *F* = 4.83, *p* = 0.0003). Female participants had lower values in sensibility threshold for interocclusal thickness when compared to males (except the initial values). However, the threshold values fall similarly in both genders after the initial measurement (Figure 2).

**Figure 3:**
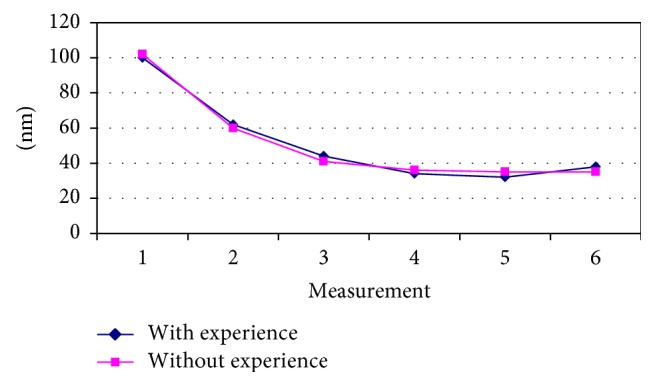
Dynamics of sensibility threshold for interocclusal thickness according to experience with complete dentures.

The denture experience factor had no significant effects in sensibility threshold for interocclusal thickness (*F* with exp. = 0 : 01, *p* = 0.9270) (Figure 3).

## 4. Discussion

The thickness discrimination test, which evaluates the ability to detect specific thickness placed between the dental arches, is among the clinical methods used to evaluate oral functions [14]. According to the objective difficulties during testing, sensibility threshold for interocclusal thickness in the region of the first-molars/premolars [14] (where tactile receptors have interference from the cheeks or tongue mucosa) may affect the analysis of the data obtained. The fact that we were interested in the period of dynamics observation of sensibility threshold for interocclusal thickness, we decided to test the positioning of the metal foil in the upper and lower incisor region in incisal edge-to-incisal-edge position teeth. The ability of the sensibility threshold is influenced by several factors, including the mechanical-physical properties of materials used for testing. Aluminum foil is the most commonly used material [15, 16].

The results of this study indicate that after insertion of complete dentures the sensibility threshold for interocclusal thickness improved due to adaptation. The sensibility threshold for interocclusal thickness shows significant variation during the observation period and in terms of the level of decline, from 88 *μ*m to 36 *μ*m, which was achieved in the fourth measurement, while in the subject with natural dentition it was 15 *μ*m. The sensibility threshold for interocclusal thickness from dynamic aspect changes in the first three measurements, where other variables that mark or indicate possible variations are not expressed. Our results support previous findings which state that the occlusal thickness perception threshold level was 30–600 *μ*m for denture wearers but 8–30 *μ*m for natural dentition [17–19].

Our findings suggest that females reach stationary state at 30% of initial values, while men reach it at 45% of the initial value of the sensibility threshold for interocclusal thickness. Additionally, the sensibility threshold for interocclusal thickness decreases significantly more in females than in males, with initial measurement difference, with women on average having higher values. Our results do not support findings by Mayers [20], because the sensibility threshold for interocclusal thickness from their methods cannot conclude in which period after insertion of the complete dentures it was developed. The fact is that a threshold for interocclusal thickness and other variables that affect the functional adaptation of the subject with complete dentures behaves in new dynamics around balance-position.

In differences of key moments of functional adaptation to new complete dentures during the observation period, this indicator has the highest rank, participating with 30%. Preliminary experience with complete dentures does not come up as an important value of the variables investigated. Otherwise, the ability to perceive thin objects between the teeth was generally better in the experienced group than nonexperienced denture wearers [8].

Implant-supported fixed dental prostheses in edentulous patients are a very invasive, expensive, long treatment option [21] but at the same time valuable treatment option for restoring edentulous patients. The authors suggest that sensory and motor feedback to the central nervous system can be restored by implant-supported full dentures consequently improving the quality of life of prostheses wearers [22–24].

## 5. Conclusions

The sensibility threshold for interocclusal thickness was the most important functional adaptation predictor in patients with complete dentures. A unique trait of this indicator is the progressive reduction of initial values and a tendency to reestablish the stationary state in the fifteenth week after dentures is taken off. The sensibility threshold for the perception of interocclusal thickness is about 30% for females and 45% for males. The results suggest that this variable can be considered as the best indicator for the functional adaptation for patients with new dentures.

## Figures and Tables

**Table 1:** Comparison of gender, age, and nonexperienced/experienced group.

	Gender		Nonexperienced/experienced group with complete dentures	
	Female	Males	Nonexperienced	Experienced
*N*	42	46	45	43
$\underline{X}$	54.6	55.7	52.7	57.8
DS	5.4	6.1	5.7	4.1
*X* max	66	68	65	68
*X* min	42	44	42	49

**Table 2:** Dynamics of sensibility threshold for interocclusal thickness.

	Gender		Nonexperienced/experienced group with complete dentures		Total
	Female	Males	Nonexperienced	Experienced	
*N*	42	46	45	43	88
1x	106	97	102	100	101
DS	1.3	2.7	2.3	2.4	1.6
2x	39	63	60	62	61
DS	1.3	2.6	2.1	2.2	1.5
3x	39	46	41	44	43
DS	1.0	1.9	1.2	2.0	1.2
4x	32	37	36	34	35
DS	0.9	1.2	1.1	1.2	0.8
5x	30	37	35	32	34
DS	0.7	0.9	1.1	0.8	0.7
6x	30	42	35	38	36
DS	0.7	0.8	1.0	6.3	3.1
